# Challenges in evaluating the accuracy of AI-containing digital triage systems: A systematic review

**DOI:** 10.1371/journal.pone.0279636

**Published:** 2022-12-27

**Authors:** Jonathan Ilicki

**Affiliations:** Innovation Team, Platform24, Stockholm, Sweden; Sreenidhi Institute of Science and Technology, INDIA

## Abstract

**Introduction:**

Patient-operated digital triage systems with AI components are becoming increasingly common. However, previous reviews have found a limited amount of research on such systems’ accuracy. This systematic review of the literature aimed to identify the main challenges in determining the accuracy of patient-operated digital AI-based triage systems.

**Methods:**

A systematic review was designed and conducted in accordance with PRISMA guidelines in October 2021 using PubMed, Scopus and Web of Science. Articles were included if they assessed the accuracy of a patient-operated digital triage system that had an AI-component and could triage a general primary care population. Limitations and other pertinent data were extracted, synthesized and analysed. Risk of bias was not analysed as this review studied the included articles’ limitations (rather than results). Results were synthesized qualitatively using a thematic analysis.

**Results:**

The search generated 76 articles and following exclusion 8 articles (6 primary articles and 2 reviews) were included in the analysis. Articles’ limitations were synthesized into three groups: epistemological, ontological and methodological limitations. Limitations varied with regards to intractability and the level to which they can be addressed through methodological choices. Certain methodological limitations related to testing triage systems using vignettes can be addressed through methodological adjustments, whereas epistemological and ontological limitations require that readers of such studies appraise the studies with limitations in mind.

**Discussion:**

The reviewed literature highlights recurring limitations and challenges in studying the accuracy of patient-operated digital triage systems with AI components. Some of these challenges can be addressed through methodology whereas others are intrinsic to the area of inquiry and involve unavoidable trade-offs. Future studies should take these limitations in consideration in order to better address the current knowledge gaps in the literature.

## Introduction

During the past years, digital online symptom checkers and digital patient-facing triage tools have become increasingly common. These tools allow patients to enter their symptoms and answer questions, and either receive possible diagnosis or advice on what level of care may be appropriate [1]. Digital triage solutions often focus on primary care conditions [2], as such conditions are often less urgent and can be triaged to various level of urgencies to optimize queues and resource allocation, and in contrast to emergency medicine triage systems, often don’t require physical examination. Artificial intelligence (AI) or machine learning is often described as a potential way to significantly improve various triage systems [3–5].

However, evaluating triage solutions is complex. It is difficult to capture the many important aspects of a triage system (e.g. such as condition coverage, diagnostic accuracy, patient safety and consequent resource utilization) with one primary outcome [6]. This complexity could explain why there are relatively few comprehensive validations of the predecessors to digital triage solutions, the traditional primary care telephone triage systems [7, 8]. Moreover, triage systems are commonly validated using patient vignettes, which are short descriptions of clinical cases with a predetermined correct diagnosis and/or level of care. Vignettes are a practical method, but might have limitations when assessing something as complex as triage.

Recent studies have attempted to compare different digital triage systems’ accuracy [9, 10]. In general, reviews conclude that studies and data on triage system accuracy remain limited despite increased usage [11]. Moreover, there is limited published research on the specific methodological challenges in studying these types of rapidly developing systems. As digital triage systems are already being implemented in healthcare [12], it is valuable to gain a better understanding of how they work.

Accuracy is a necessary, but not sufficient, criteria for a triage system to be useful. Understanding potential limitations in understanding triage accuracy with vignettes could be useful, considering the potential mismatch between using standardised vignettes to assess a complex intervention. A better understanding of the specific challenges in studying digital AI-based triage systems’ accuracy could be useful when designing future studies. This systematic review therefore aims to summarize the current knowledge regarding obstacles for studying digital patient-operated AI-based triage systems’ accuracy in a primary care setting.

## Materials and methods

This systematic review was carried out in accordance with PRISMA guidelines (PRISMA checklist available as S1 Checklist) [13]. No predefined or preregistered protocol was used. The research question was: What limitations exist for studying the accuracy of digital patient-operated AI-containing triage systems for primary care? This question was deconstructed using the PICO structure [14] in order to design the search strategy. As studies typically mention methodological limitations in their discussions, a PICO was constructed to identify primary studies on the topic, with the goal of later synthesizing all limitations identified in the various studies. Consequently, the PICO focused on identifying studies containing such limitations. The deconstructed form of the question was: what are the limitations found when studying [Outcome:] accuracy with regards to appropriate urgency/level of care for [Population:] primary care patients with all types of conditions when [Intervention:] triage assessment is performed by digital patient-operated triage system in comparison to [Comparison:] regular triage systems utilized by healthcare staff?

### Search strategy

A literature search was performed on the 10^th^ of September 2021 in the following databases: Pubmed (NCBI), Scopus (Elsevier) and Web of Science (Clarivate). The search string was iteratively designed due to an initial paucity of results, finally only incorporating the population and intervention components of the PICO in order to minimize the risk of missing relevant studies. The following search phrase was ultimately used, and MeSH terms used for searching PubMed were adapted for searches in Scopus and Web of Science.

*((triage OR “symptom checker”) AND ("artificial intelligence"[MeSH] OR “machine learning”[MeSH] OR “AI” OR “neural network” OR “supervised learning” OR “NLP”)) AND ("Primary Health Care"[MeSH] OR "General Practice" OR “GP clinic” OR “primary care”)*

No language restrictions were applied during the search and retrieval of articles. Databases were searched from inception.

#### Selection criteria

Following removal of duplicates, the remaining article titles and abstracts were screened by the author. Full articles, abstracts, pre-prints, and posters were included with no restriction on date of publication. All study types were included with no restriction. Articles were included for full text review if their abstract described a digital triage or symptom checking system. During full text review, articles were excluded from data extraction if they did not include a system able to be used in a primary care setting (i.e. handle a general population), were limited to triaging only a specific condition or specific group of conditions, were not patient-operated, did not report accuracy or if the studied system did not have an AI component. All types of AI were included, regardless of if the component was patient-facing or not.

**Fig 1:**
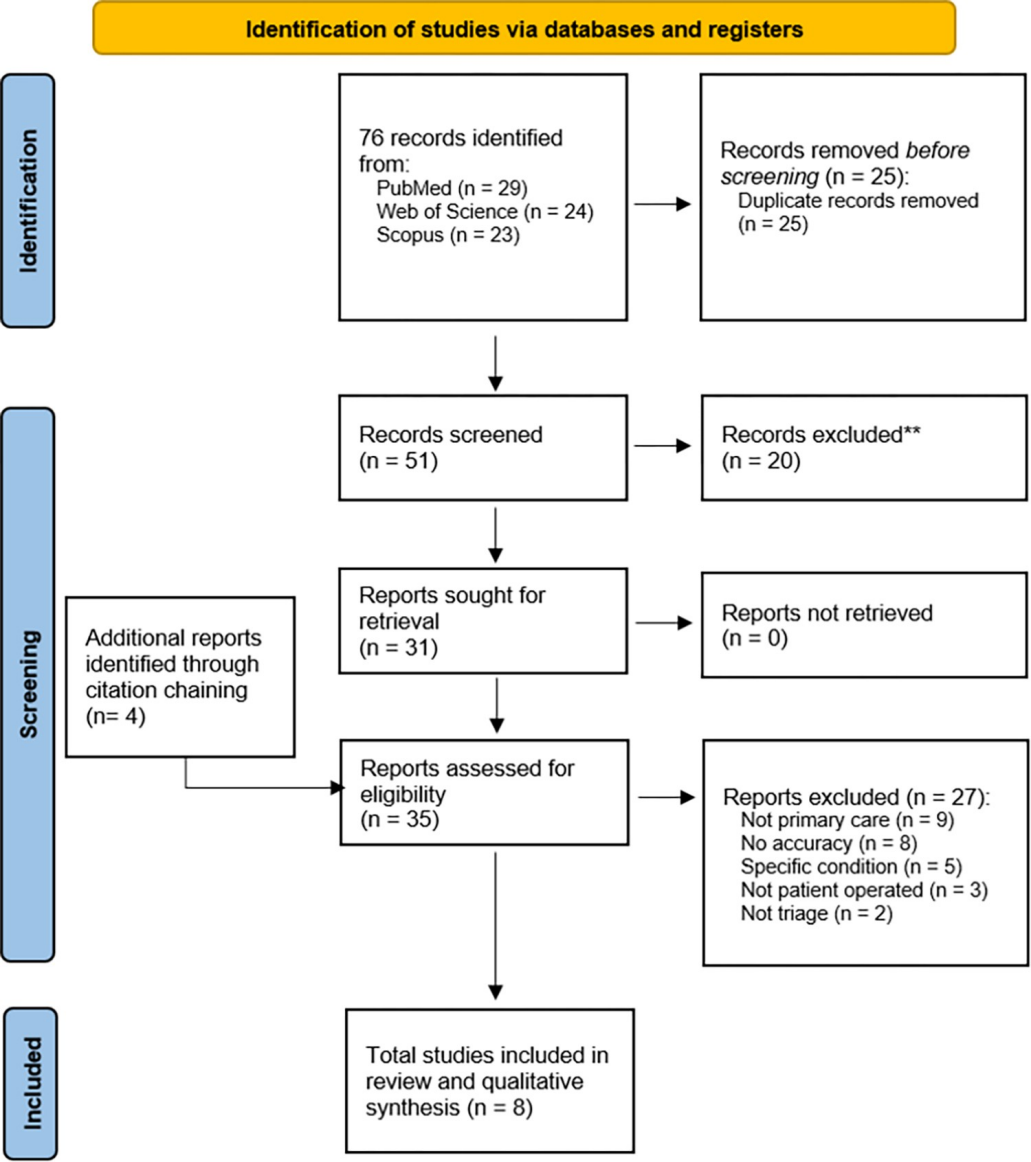
PRISMA flow diagram.

To widen the search and find other articles that might be relevant, articles’ references were searched for potentially relevant articles (i.e. citation chaining), and these articles were retrieved from PubMed and added to the results. No automation tools were used. The search and selection PRISMA flow chart is depicted in Fig 1 [13].

### Data extraction and synthesis

Data from the included studies were extracted as per Table 1 below:

**Table 1:** Extracted data from included studies.

Category	Data type
Context	Study design
	Study population
	Study country
	Any Author COI
Study	Study objective
	Method of studying accuracy
	Definition of triage accuracy
	Gold standard for triage accuracy
	Any other comparator
	Number of interactions for testing triage system
Results	Reported accuracy
	Main limitations mentioned in article
Triage system	Triage system name
	Type of AI component

If data was missing for a variable this was denoted as “not available”. Retrieved articles were too heterogenous to be synthesized quantitatively and were instead qualitatively synthesized. Quality assessment tools for assessing potential bias were deemed less useful as this review assessed limitations in articles, and not the studies actual results. Each article was assessed by the author and no automation tool was used. Main limitations were defined as explicit mentions of limitations or challenges, which relate to the study’s ability to address the question of accuracy. Other limitations (e.g. relating to statistical features or patient recruitment) were not included.

The limitations described in all included studies were synthesized using a qualitative thematic analysis. This analysis entails a qualitative analysis of the findings, and an iterative grouping in order to identify subthemes and overarching main themes. All included studies were included in the thematic synthesis.

## Results

The literature search yielded 76 articles, from which 25 duplicates were removed. The abstracts of the remaining 51 articles were screened. 20 of these 51 were not included as they did not meet inclusion criteria. The remaining 31 articles went through abstract and full text review. Of these, 27 were excluded per the abovementioned criteria, leaving 4 articles. These 27 excluded articles were excluded as they

- did not address a primary care setting (n = 9) [15–23],
- did not report accuracy (n = 8) [24–31],
- studied a system not triaging broad range of conditions (n = 5) [32–36],
- did not study a patient-operated system, (n = 3) [37–39],
- were not triage related (n = 2) [40, 41].

The references of all full-text reviewed articles were searched for relevant articles and through this citation chaining an additional 4 articles were identified. Thus, a total 8 articles were found to be relevant to the research question and were grouped into 2 categories:

1. Primary studies on one or several digital triage systems’ accuracy (n = 6) [1, 10, 42–45]
2. Reviews which include assessments of digital triage systems’ accuracy (n = 2) [11, 12]

The included primary studies are summarized in Table 2 below.

**Table 2:** Primary studies reporting studies and limitations on determining digital triage systems’ accuracy.

Year, Author	Study design	Study population (# of digital systems)	Study country	Author COI	Study Objective	Method of studying accuracy	Definition of triage accuracy	Gold standard for triage accuracy	Any other comparator	Number of interactions for testing AI triage system	Reported accuracy	Main limitations described in article	Triage system name	Type of AI^†^
2015, Semigran*	Audit study	23, of which 3 offered triage advice and *have AI component*	USA	No	Determine diagnostic and triage accuracy of online symptom checkers	45 self-designed vignettes; common and uncommon conditions in the US	Assess if the system recommended emergent care, non-emergent care, or self care	Predefined correct acuity	No	45 per system	Appropriate in 57% of cases	• Unsure if all symptom checkers were tested • Vignettes with clear symptoms and diagnosis • Specific clinical language not representative of how systems will perform when used by patients • vignettes not tested with physicians • Highest acuity was chosen when triage system suggested several care sites	Isabel; Symptify; Symptomate	NLP [46];
														NLP [47]; NLP+BM [48, 49]
2016, Middleton	Commentary (not peer-reviewed)	1	UK	Yes	Describe how a digital AI-triage system can be developed and validated	102 self-designed vignettes by external physician	Assess recall, precision, accuracy, safety of system	Three expert clinicians	12 GPs, 17 nurses	102	Accurate in 90% of cases. Safe in 100% of cases.	• None clearly stated	Babylon	NLP+BM [44]
2018, Ghosh	Commentary	1	Australia	Yes	Demonstrate development of automated chatbot	30 self-designed vignettes; 10 emergent care, GP, and self-care.	Assess if (1) if at least one of t top 3 reported conditions was correct (2) if 2 of 3 reported conditions were correct	Inhouse specialists	No	30	Accurate in 67–83% of cases	• None clearly stated	Quro	NLP [43]
2018, Razzaki	Experimental (not peer-reviewed)	1	UK	Yes	Compare AI powered triage system’s triage and diagnostic accuracy with that of GPs	100 vignettes created by independent medical practitioners; common and rare conditions	Assess (1) safety: recommendation of equal or greater urgency than judge’s minimum triage. (2) appropriate triage: recommendation within judge’s range of acceptable recommendations	One senior medical practitioner	6 GPs	100	Safe in 97% of cases. Appropriate in 90% of cases	• Included rare conditions, not reflecting real world accuracy • Can only compares cases without physical examination • Cases using only one condition • High interrater variability between physicians	Babylon	NLP+BM [44]
2020, Entezarjou	Experimental	1	Sweden	Yes	Determine interrater variability between machine learning triage and human triage	276 real-life medical history reports covering 10 most common chief complaints in the digital platform	Assess if algorithm correctly determined a need or no need of an urgent physical examination	Majority vote of 5 GPs	No	276	Correct for 74% of nonurgent cases; 42% of urgent cases	• Sample not representative of physical primary care population • No out-of-sample external validation • Defining “correct” triage prospectively may be impossible as some clinical outcomes cannot be predicted • Voting used, and lack of consensus for gold standard • Various methods of defining gold standard makes comparison with other studies difficult	-	BM [45]
2020, Gilbert*	Experimental	8, of which 7 offered triage advice and have AI *component*	Germany	Yes	Compare condition coverage, accuracy of suggested conditions and appropriateness of urgency advice	200 self-designed vignettes; common and less-common primary care conditions	Assessed proportion of ‘safe’ urgency advice: at gold standard level, more conservative, or no more than one level less conservative	3 external GPs	7 GPs	200 per system	Different across systems: coverage 52–99%, accuracy 32–82%. safety 80%-98%.	• Clinical vignettes rather than real-patient data • Real patient data as data source is problematic, requiring physical consultation to confirm diagnosis • It has been argued that lay-person entry is closest to the real intended use of symptom assessment apps; however, it is known that lay-people are less reliable at entering clinical vignettes than healthcare providers. • Non-systematic/comprehensive process to select apps to test • Some systems don’t provide triage advice for certain conditions • Potential UK bias in vignettes, some systems mainly used in USA • Risk that vignette is phrased in more accessible manner for apps than average patient presentation • Software evolves rapidly, and performance can change since data collection • Researchers include employees of manufacturers	Ada; Babylon; Buoy;	NLP+BM [50, 51];
														NLP+BM [44];
													K Health; Mediktor; Symptomate; Your.MD	NLP+BM [52];
														NLP+BM [53];
														NLP [54];
														NLP+BM [48, 49];
														NLP+BM [55]

AI = Artificial Intelligence. BM = Bayesian model. COI = Conflict of interest. GP = General Practitioner. NLP = Natural Language Processing. *Only data for systems with AI component and offering triage advice are described in table above, why some systems reported in Semigran et al and Gilbert et al are excluded. ^†^Detailed information on specific AI components was not available for all systems, and this list may therefore not be completely exhaustive.

The two reviews retrieved in the search process were both systematic reviews assessing several aspects of digital and online symptom checkers and similar services [12], and intelligent online triage tools [11]. Both reviews were searched for additional primary that fulfilled this study’s inclusion criteria, but none were found. The reviews are summarized briefly in Table 3.

**Table 3:** Summary of retrieved reviews.

	Chambers 2019	Gottliebsen 2020
Study design	Systematic review with narrative synthesis	Systematic literature review with thematic analysis
Objective	Review evidence on digital and online symptom checkers and similar services	Explore features of intelligent online triage tools in primary care
Number of included studies	27 studies, of which 2 studies included patient-operated triage systems with AI-components [1, 42]	17 articles, of which 3 studies included patient-operated triage systems with AI-components [1, 43, 44]
Data sources	Medline, Embase, Cochrane Library, CINAHL, Health Management Information Consortium, Web of Science and ACM Digital Library—up to April 2018	PubMed, Cochrane library—up to March 2019
Conflict of interest	No	No
Reported accuracy	• Inconsistent findings for accuracy of triage; • Performance variable between different systems • Mixed picture but most symptom checkers were inferior and/or more cautious in their triage advice	• Triage advice from the symptom checkers was generally risk averse and inappropriate for many of the vignettes.
Examples of limitations highlighted in articles	• Regular updating needed to keep track of new studies • Users in study not representative of typical users • Covering limited range of conditions • Using doctors’ clinical diagnosis as gold standard	• Clinical vignettes not comparable to complexity of advising patient using natural language • Treats primary care contexts in Western countries homogeneously

The limitations described in all included studies were then synthesized using a qualitative thematic analysis, which is described in Table 4.

**Table 4:** Themes of the limitations described by authors in retrieved articles.

Main theme	Subthemes	Limitation
1. Ontological limitations in studying heterogenous novel technology	1. A rapidly developing field	• Regular updating of review needed to keep track of new studies [12] • Software evolves rapidly; performance can change since data collection [10]
	2. There are many different digital triage systems	• Unsure if all symptom checkers were tested [1] • Non-systematic/comprehensive process to select apps to test [10] • Some systems don’t provide triage advice for certain conditions [10]
	3. Intervention is context-dependent	• Treats primary care contexts in Western countries homogeneously [11] • Potential UK bias in vignettes, some systems mainly used in USA [10]
2. Epistemic limitations in studying triage accuracy	1. No simple way to agree upon gold standard as triage has intrinsic uncertainty	**Difficult to define gold standard** • Lack of consensus during vote used to define gold standard [45] • High interrater variability between physicians [44] • Defining “correct” triage maybe impossible, if some outcomes can’t be predicted [45] • Vignettes not tested with physicians [1] **Various ways to define gold standard** • Various methods of defining gold standard, difficult to compare studies [45] • Doctors’ diagnosis as gold standard biases comparison in favor of doctors [12]
3. Methodological limitations due to methodology of vignettes	1. Vignette design lacks external validity	**Vignette content/mix not representative of real life** • Vignettes not representative of physical primary care population [10, 45] • Vignettes covering limited range of conditions [12] • No out-of-sample external validation [45] • Vignettes with clear symptoms and diagnosis [1, 44] **Vignette wording not representative of real life** • Vignette phrasing potentially more app-friendly than average patient presentation [10] • Performance with clinical language not representative of performance with patient usage [1] **Vignettes don’t assess impact of patient interaction with system** • Triage system can recommend several care sites: highest acuity was assessed [1] • Clinicians (not lay-persons) entered vignettes [10] • Clinical vignettes not comparable to advising real patient using natural language [11]
	2. Using real patient data rather than vignettes	• Using real patient data is problematic; either physical consultation needed to confirm diagnosis [10], or can only compares medical cases that don’t need physical examination [44]
4. Limitations due to conflicts of interest	1. Industry research	• Researchers include employees of manufacturers [10]

## Discussion

The synthesis of the articles identified in this systematic review revealed several themes which are relevant to studying the accuracy of digital triage systems.

### Ontological limitations in studying rapidly developing and highly contextual novel technology

Several studies highlighted that there is an intrinsic challenge in studying a rapidly developing field [1, 10, 12]. It can be difficult to assess a heterogeneous group of AI-powered digital triage systems as new software is developed and existing software is updated continuously, as systems can differ from each other, as new systems might arise and as the performance of systems can change over time as software is updated. Assuming that an inductively studied phenomenon will not change over time is a well-known problem of induction, as extensively discussed by e.g. Karl Popper [56]. In this specific case, this entails a limitation in the external validity one can expect when studying rapidly developing technological fields.

The identified studies also highlight that studies assessing the accuracy of triage systems treat primary care contexts homogenously [11]. This can be an issue as systems and/or vignettes can have a geographical bias [10], in e.g. what conditions are common or regarding how urgent certain conditions are deemed to be. Different countries have different healthcare systems and often use different triage solutions, as exemplified by many countries using a triage system developed in that country. The results of a study on a digital triage system in a specific context might not be representative of the system in a different context. Both of these limitations relate to the ontology of a rapidly developing and highly contextual intervention such as digital triage software. The limitations are difficult to mitigate through study design, and should be kept in mind when assessing studies on such interventions.

### Epistemological limitations in studying triage accuracy

Some studies discuss the limitations in defining a gold standard for what is appropriate or safe triage [12, 44, 45]. All retrieved studies used a selected group of clinicians’ assessments to define a gold standard. The validity of this can be questioned due to high interrater variability [44, 45], lack of consensus [45], varying methods across studies [45], and that this definition inherently biases the assessment in favor of clinicians [12]. However, it is not clear what alternative method one could use instead. Furthermore, there is an challenge as some outcomes might not be able to predict in advance [45], and such cases will be often excluded from vignette testing.

First, this highlights that there is no universal consensus on what is appropriate triage, and how triage systems’ accuracy should be tested. Triage entails assessing a patient’s medical needs with less information than would be obtained during a consultation. Triage will therefore always involve some level of tradeoff between decreasing resource utilization and increasing the risk for missing pertinent clinical information that might affect the assessment of the patient. Reaching a consensus on what is appropriate triage outcomes is not possible without an underlying consensus on what level of risk one is willing to accept and what level of resource utilization is optimal. Moreover, as long as there are various opinions on what tradeoff is optimal, it will be difficult to compare studies on different systems in different contexts.

Second, high interrater variability has been observed in other triage studies. Studies on emergency triage have demonstrated that interrater variability can be high when applied by clinicians [57, 58], and that triage scales seldom consistently show high reliability [59]. This illustrates a methodological tradeoff when studying triage accuracy: between using simple patient vignettes with one clear diagnosis, in which interrater variability most likely will be lower, and more real-life cases, where interrater variability most likely will be higher. Both alternatives have limitations, either limiting the external validity or the reliability of the comparator (internal validity).

### Methodological limitations in using patient vignettes

All primary studies used vignettes to assess triage accuracy, and several discuss associated methodological limitations, primarily related to external validity. Some of these limitations are addressable through adjusting vignette design, whereas others are more difficult to mitigate. These limitations and potential mitigations are described in Table 5 below.

**Table 5:** Challenges in external validity.

Limitation	Examples from included articles	Possible mitigations
**Vignette content/mix not representative of real life**	• Vignettes not representative of physical primary care population [10, 45] • Vignettes covering limited range of conditions [12] • No out-of-sample external validation [45] • Vignettes with clear symptoms and diagnosis [1, 44]	Design vignettes based on • real-life cases, • real-life case mix, • with wide range of conditions, • where final diagnosis is known
**Vignette wording not representative of real life**	• Vignette phrasing potentially more app-friendly than average patient presentation [10] • Performance with clinical language not representative of performance with patient usage [1]	• Design vignettes using patient language • Let laypersons enter vignettes into system to better mimic real-life usage [60]
**Vignettes don’t assess impact of patient interaction with system**	• Triage system can recommend several care sites: highest acuity was assessed [1] • Clinicians (not lay-persons) entered vignettes [10] • Clinical vignettes not comparable to advising real patient using natural language [11]	Use lay-persons to test vignettes and also select among triage systems’ final recommendations

The possible mitigations described in Table 5 could potentially improve the external validity of future vignette studies on the accuracy of digital triage systems. However, some of the limitations are more difficult to address.

First, vignettes with clear symptoms and diagnosis can differ from real life cases which can have more complex and ambiguous presentations. However, vignettes with more complex and ambiguous cases (i.e. with higher external validity) will more likely suffer from a higher interrater variability when defining a gold standard. This tradeoff between external and internal validity becomes more complex considering that vignette case mix should be adjusted for specific healthcare contexts. By adjusting the case mix to better reflect a certain geography or practice, one will unavoidably decrease it for other geographies or practices. Moreover, if cases are weighted so that e.g. common or dangerous cases are overrepresented, then the researchers’ choice of allocating weights can greatly affect the results [44].

Second, clinical vignettes presented to a digital system that recommends a certain triage outcome is a different phenomenon than a human advising a real patient with natural language [11]. Even if a triage system had a perfect accuracy, certain things will be lost (e.g. the social interaction between a patient and a clinician) and some things will be gained (e.g. removing the risk that practitioner gender affects the triage assessment [61]).

Finally, as highlighted by some studies, using real patient data instead of constructed vignettes can be challenging [10, 44]. Either one must select cases which don’t need a physical examination (limiting what cases one test the system with) or one includes cases in which a diagnosis was obtained using a physical examination (information which the triage system will not be given). Previous research has analyzed the various aspects of vignettes, comparing construct, internal and external validity, as well including the strengths and weaknesses of using clinical vignettes [62]. Unfortunately, recommendations given regarding vignette content do not address the challenges discussed above.

### Limitations due to conflicts of interests

Five of the 6 identified primary studies had authors which declared a conflict of interest [10, 42–45]. Industry sponsored device studies have a risk for bias [63], and report more positive efficacy results and favorable conclusions than non-industry sponsored studies. Two of the primary studies are non-peer reviewed preprints that have not since been peer-reviewed and published [42, 44]. Certain methodological weaknesses in one of the preprints has since been raised [64, 65].

### Implications for practice and future research

This review identified certain themes of limitations which impact the ability to assess the accuracy of digital AI-containing patient-facing triage systems. The identified limits as well as possible mitigations are summarized in Table 6 below.

**Table 6:** Possible mitigations based on synthesis of identified limitations.

Limitation group	Possible mitigations for researchers
Ontological limitations in studying heterogenous rapidly developing technology	• Be explicit on what system is being studied, in what geography, and in what context, to facilitate clinicians in assessing studies’ transferability to other digital systems, one’s specific healthcare context when assessing such studies as well as one’s specific patient case-mix.
Epistemological limitations in studying triage accuracy	• Be explicit about the challenges in defining a gold standard for triage • Be explicit about the rational for choosing simple cases (for which a gold standard is easier to define) or complex cases (which better represent real life). • Use more clinicians or existing guidelines for defining a gold standard triage outcome/range
Methodological limitations related to use of clinical vignettes	• Design vignettes based on ○ real-life primary care cases ○ real-life case mix, ○ with wide range of conditions, ○ where final diagnosis is known • Design vignettes using patient language and let laypersons enter vignettes into system, and reach final triage recommendation • Consider other non-vignette methodology
Conflicts of interest	• More research is needed by independent researchers • Healthcare providers using such systems, and system developers should facilitate researcher’s access to such data

The ontological limitations aren’t addressed by the recommendations above, but the mitigations align with clearer reporting of important health informatics principles, and can be achieved by e.g. adhering to STARE-HI guidelines [66].

Fraser *et al* have published a suggested guideline with five sequential steps for evaluating symptom checkers [65]. Some of their recommendations overlap with those above, e.g. that vignettes can have higher external validity by including common and dangerous conditions. Several of the methodologies they recommend, including observational trials and RCTs, overcome the limitations of using vignettes. However, other researchers that also emphasize the need of RCTs argue that they should only be undertaken when the software is stable (so that future changes will only be minor) [67]. This is challenging for software that is continuously being developed. Other of Fraser *et al*’s recommendations, such as routine and random auditing of cases once a system is used, may partially address this, but may also face challenges in the heterogeneity of the contexts and systems.

In summary, this systematic review of studies on the accuracy of digital triage systems uncovers several methodological improvements which future researchers could consider, as well as epistemological and ontological limitations which challenge what knowledge can be obtained regarding such systems using such methodologies. This does not mean that studies on triage systems should not be performed, but rather that more studies are needed, and that decision-makers and clinicians should be aware of non-methodological limitations when assessing this literature.

### Limitations and strengths

This review importantly and self-referentially has numerous limitations which are important to keep in mind when interpreting the results. First, despite a broad search strategy with few restrictions and citation chaining a limited number of articles were found. Pertinent studies may have been missed in other databases not searched, grey literature or other languages. Similarly, this is a rapidly developing field and it is probable that new studies will have emerged when this is being read and that these studies could change this reviews results. Second, the review was not preregistered and only one author with a conflict of interest assessed the articles. Third, the study’s research question was limited to digital systems containing AI-components. The review therefore excluded studies not containing AI, which may indirectly have been relevant to the research question. Fourth, this systematic review can describe limitations mentioned in retrieved studies, but does not address limitations identified by end-users and clinicians that implement such systems. Including that perspective would be useful for creating recommendations for how to better design such studies. Fifth, the limitations identified in this review, and potential methods of mitigating them, were compared to existing literature on e.g. reporting on health informatics studies. As no systematic review was performed on methods to address the epistemological, ontological or methodological challenges, there are most likely other studies and frameworks not identified in this study, which could be useful in mitigating the limitations.

This review also has several strengths. First, other recent reviews did not include any primary study not identified in the search, which implies that this search strategy is less likely to have missed significant amounts of relevant literature. Second, to the extent of the author’s knowledge, this is the first comprehensive study on the challenges in studying the nascent field of digital AI-containing triage systems, and identifying these challenges may assist future researchers in decisions regarding study design. Finally, certain limitations which are difficult for researchers to address are highlighted, so that clinicians critically appraising the literature can better understand and assess such studies.

## Supporting information

S1 Checklist PRISMA 2020 checklist. (DOCX)
